# The Efficacy of Curcumin Patch as an Adjuvant Therapeutic Agent in Managing Acute Orofacial Pain on the Post-Cleft Lip and Cleft Palate Surgery Patients: A Pragmatic Trial

**DOI:** 10.1055/s-0042-1750802

**Published:** 2022-09-12

**Authors:** Tantry Maulina, Yohanes Yoppy Purnomo, Salshabia Gabrielle Raissa Tasman, Endang Sjamsudin, Amaliya Amaliya

**Affiliations:** 1 Department of Oral and Maxillofacial Surgery, Faculty of Dentistry, University of Padjadjaran, Bandung, Indonesia; 2 Oral and Maxillofacial Surgery Department, Faculty of Dentistry, University of Padjadjaran, Bandung, Indonesia; 3 Department of Periodontology, Faculty of Dentistry, University of Padjadjaran, Bandung, Indonesia

**Keywords:** curcumin patch, orofacial pain, cleft lip surgery, acute pain, postoperative pain, activity, cry, consolability, cleft palate surgery

## Abstract

**Objective** Acute pain is one of the most common pains experienced by post-cleft lip or cleft surgery patients regardless of the administration of analgesic agents. This current study aimed to evaluate the efficacy of a curcumin patch as an adjuvant analgesic agent on the post-cleft lip and cleft palate surgery patients.

**Materials and Methods** Fifty-five (33 male; 22 female) participants aged 36 months or less are recruited in this pragmatic trial and randomly assigned to a control group, where no curcumin patch was applied; or the experimental group, where the participants wore a curcumin patch with a dosage of 100 mg. All participants (regardless of the group) received a standardized postsurgery analgesic agent immediately after the surgery was completed. A face, leg, activity, cry, and consolability (FLACC) scale was used to evaluate pain levels for three subsequent time points.

**Statistical Analysis** All data were then analyzed by using the Mann–Whitney U test to compare the mean differences between the two groups.

**Results** The results of the current study revealed that there was no significant difference found between the control and the experimental group when mean pain scores were compared for the first evaluation time. Yet, there was a significant difference ( *p* < 0.01) between the two groups' mean pain scores on the second evaluation time.

**Conclusion** Curcumin patch was found to be effective when used as an adjuvant analgesic agent to reduce acute-orofacial postsurgery pain in cleft lip and cleft surgery patients.

## Introduction

The latest meta-analysis study performed by Salari et al about the prevalence of cleft lip, cleft palate, as well as cleft lip and palate, revealed that the prevalence of cleft palate based on the 59 studies included in the meta-analysis was 0.33 in every 1,000 live birth (95% confidence interval [CI]: 0.28–0.38); the prevalence of cleft lip based on the 57 studies included was 0.3 in every 1,000 live birth (95% CI: 0.26–0.34); and the prevalence of cleft lip and palate based on 55 studies included was 0.45 in every 1,000 live birth (95% CI: 0.38–0.52), indicating the high prevalence of these birth defects 1 and their common occurrence in human beings. 2 Aside from its high prevalence, patients and/or family member of patients with cleft lip and/or cleft palate also reported impacted quality of life. 3 4

Considering the high prevalence as well as the impact on patients' quality of life, the treatment of cleft lip and/or cleft palate is performed early in life. The course of treatment for cleft lip and/or cleft palate patients consists of several stages, with surgery being the first corrective procedure to go through. 5 6 It is important to conduct surgery as early as possible as it is crucial to make the patient has a (close to) normal anatomical structure that can function as (near to) normal as possible. 7 Unfortunately, similar to any other invasive approach, surgery has postoperative consequences, one of which is postoperative pain, regardless of the existing postoperative pain management. 8 9 10 In a study conducted by Augsornwan et al, it was reported that 48% of patients who underwent palatoplasty have moderate-to-severe postoperative pain at the 4th hour, 8 indicating the importance of adequate pain management, considering that patients of labioplasty (corrective surgery of cleft lip) and palatoplasty (corrective surgery of cleft palate) are mainly very young children that cannot properly communicate their pain level (yet).

Unlike postoperative pain control in adult patients that can involve the usage of opioid analgesics, the usage of opioid analgesics in infants who underwent surgery has been reported for its disadvantages, namely postoperative sedation, respiratory depression, as well as consequent airway compromise. 9 11 Therefore, various combinations of methods have been applied to reduce this postoperative pain experienced by postlabioplasty and postpalatoplasty patients, namely the addition of local anesthesia procedure to the standardized anesthesia procedure, the usage of opioids and the usage of nonsteroid anti-inflammation drugs. 9 11 12 13 Yet, regardless of these various attempts, no single postoperative pain control procedure for postlabioplasty and postpalatoplasty has been strongly recommended, which might be due to the various results.

Considering that most of the current postoperative pain control methods consist of invasive procedure(s) with possible additional side effects, ongoing research that evaluates the efficacy of postoperative pain control methods for postlabioplasty and postpalatoplasty patients is currently taking place, including the ones that involve the utilization of natural ingredients. One of the natural ingredients that have been widely evaluated and acknowledged for its analgesic effect is curcumin. 14 15 16 17 Based on these previous findings, a study that aimed at evaluating the analgesic effect of curcumin was designed. Therefore, the current study aimed to evaluate the effectiveness of a curcumin patch as an adjuvant analgesic agent in managing acute postoperative pain in postlabioplasty and/or postpalatoplasty patients. Considering that the patient will have a postoperative wound in the oral and facial area, the curcumin patch will be placed on the patient's chest.

## Materials and Methods

Fifty-five (33 male: 22 female) participants aged 36 months old or younger that went through corrective surgery for a cleft lip or cleft palate at Unpad Dental Hospital in Bandung, Indonesia, were recruited for the current study. Prior to the start of the study, ethical clearance was gained from the Universitas Padjadjaran Research Ethics Committee (No. 715/UN6.KEP/EC/2020). To confirm, every procedure and ethical aspect of the current research has been conducted in full accordance with the World Medical Association Declaration of Helsinki. All participants signed informed consent to consent to their participation and any future scientific publication as the result of their participation in the current study.

### Inclusion and Exclusion Criteria

Participants that fulfilled the following inclusion criteria (1) who had completed the cleft lip and/or cleft palate surgery procedure; (2) aged 36 months or less; (3) had no allergic history to curcumin; (4) had an initial pain score of 3 or greater than 3 on a scale of 0–10; (5) did not have any injuries at other parts of the body that have the potential to cause pain, aside from the postoperative wounds, were recruited. Those who (1) consumed additional pain or anti-inflammatory medication in addition to the curcumin patch or the standard analgesic medication prescribed by the doctor in charge and (2) removed the curcumin patch position from the initial location given by the investigator during the study period were excluded from the study.

### Sample Size Calculation

The sampling in this study was performed by using a nonprobability sampling technique with a purposive sampling type. The sample size calculation is performed using the following formula:







### Information

n1 = n2 = sample size

Zα = type I error = 5% (Zα value based on Z table is 1.64)

Zβ = 20%, type II error, (the Zβ value based on the Z table is 0.84)

X1- X2 = minimum difference which is considered significant.

S = Because there is no data regarding the standard deviation of the mean difference between patients receiving curcumin and those receiving standard drugs, the researchers suspect that the standard deviation is twice the minimum of the mean difference that is considered significant = 2×10 = 20.

Based on the above calculations, the number of participants for each group is 25 participants.

### Curcumin Patch

**Fig. 1:**
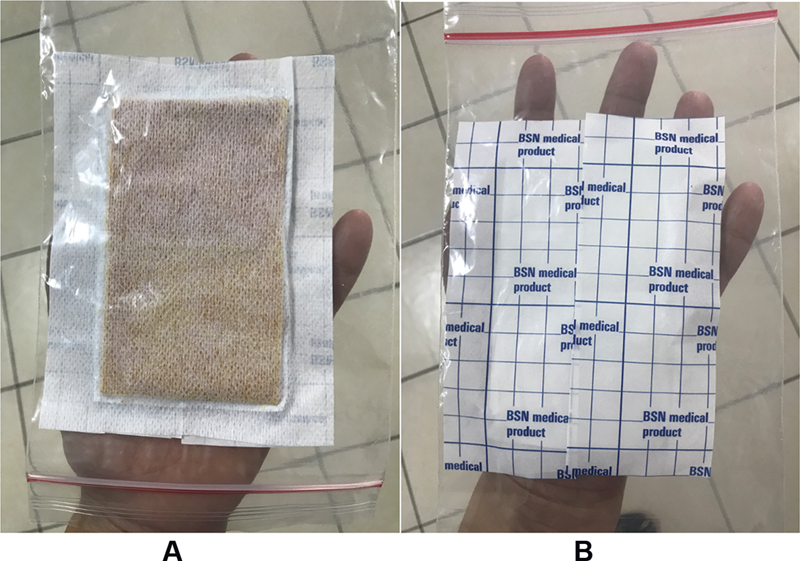
A 100 mg curcumin patch sized 6×10cm used as an adjuvant analgesic agent to manage acute post-surgery orofacial pain.

**Fig. 2:**
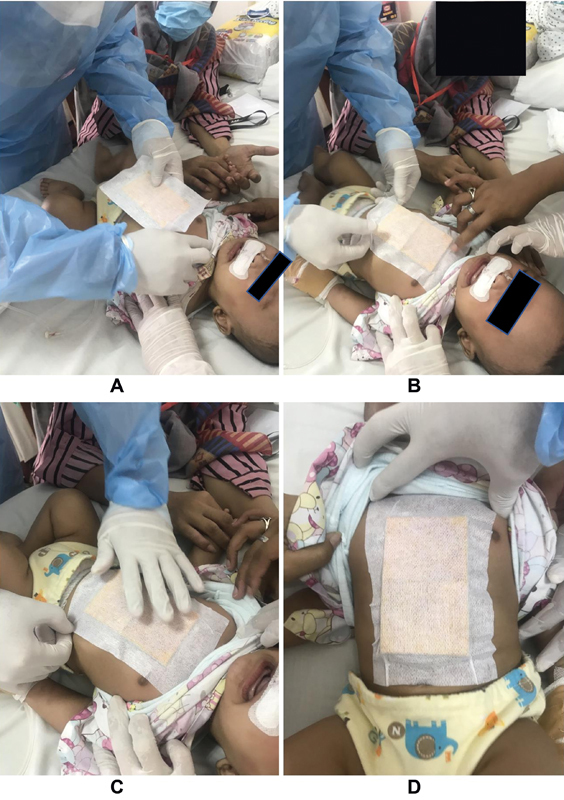
Curcumin patch placement procedure (A-B-C-D subsequently).

The curcumin patch was formulated from a mixture of curcumin extract (obtained from an Indonesian national brand Sidomuncul), hydroxypropyl methylcellulose, ethyl cellulose, polyvinylpyrrolidone, Nipagin, Nipasol, Tween 80, and 95% ethanol. The curcumin patch was in a form of a 6cm×10cm patch ( Fig. 1 ). Every patch contained 100 mg of curcumin and was prepared based on the procedure described in the previous study. 18 Once the anesthesia effect wore off, our field investigator applied the curcumin patch to the chest area of the participant ( Fig. 2 ). The patch was applied for 8hours before it was removed.

### Study Design

All participants in this study followed the postsurgery standardized operational procedures (SOP) for cleft lip or cleft lip surgery patients. According to the SOP, once the surgical procedure was completed, participants who weighed more than 10kg received 100 mg suppository ketoprofen, while participants who weighed less than 10kg received 50 mg. The next dose of ketoprofen was registered 12hours after the first dose.

### Randomization and Blinding

Once the participants completed the operation and received the standard postsurgery analgesic agent, initial pain evaluation by the #1 and #2 field researchers was performed. Participants were then randomly assigned to the control group, where the participants did not receive a curcumin patch; or the treatment group, in which the participants received a 100 mg curcumin patch. The #3 field researcher that was assigned for curcumin placement took a sealed envelope that contained the name of the group the participant was assigned to. Once revealed, this #3 field researcher made a note about which group the patient was assigned to and performed the patch placement procedure. Therefore, the field researchers who were assigned to perform the pain evaluation did not have the knowledge of which group the participant was assigned to.

### Pain Evaluation

The pain evaluation was performed by two field researchers simultaneously by using the face, leg, activity, cry, and consolability (FLACC) pain scale. The FLACC is a scale that consists of five subsections that are used to assess pain in children aged from 2-month-old to 7-year-old who are unable to communicate their pain. It has been validated and tested for reliability and validity in several previous studies. For each subsection, the pain level is scored as 0, 1, or 2. Therefore, the lowest total scoring would be 0 (zero), while the highest is 10 (ten). Further explanation of the FLACC and the scoring system can be viewed in previous studies. 19 20

### Data Analysis

Prior to the main analysis, all data were analyzed by the Kolmogorov–Smirnov normality test. The data were then analyzed by the Mann–Whitney U test to compare the mean differences between the two groups.

## Results

This current study recruited 55 participants aged between 0 and 36 months old, where most of the participants are males aged between 0 and 18 months ( Table 1 ). Most of the participants went through a labioplasty procedure. Three participants went through a labiopalatoplasty procedure, which means the participants underwent two procedures (labioplasty combined with palatoplasty) at once. Out of these three participants, two participants were in the experimental group, and one participant was in the control group.

**Table 1:** Demographical and clinical characteristics of the participants

Variable	Number of participants ( *n* )		
Sex	Male		Female
	33 participants		22 participants
Age	0–18 months		19–36 months
	36 participants		19 participants
Type of operation	Labioplasty	Palatoplasty	Labiopalatoplasty
	33 participants	19 participants	3 participants
Group	Control		Treatment
	28 participants		27 participants

Pain evaluation by using FLACC revealed that the mean pain score immediately after the general anesthesia wore off (T0) for both groups from a scale of 0 to 10 was 8.02 (standard deviation [SD] =  1.99), while the pain score in the control group for this evaluation point was 7.54 (SD =  2.12) and the experimental group was 8.52 (SD =  1.76) ( Table 2 ). The application of the curcumin patch in the experimental group resulted in significant pain score reduction, which resulted in a mean pain score of 2.48 for the 8th hour postoperative pain evaluation (T2). Although the control group also showed pain reduction, the T2 pain score for the control group was still higher than those of the experimental group.

**Table 2:** Comparison of the pain score between the control group and the treatment group by using FLACC pain scale

Group	Minimum	Maximum	Mean	SD
Both				
T0 (immediately after anesthesia wore off)	3	10	8.02	1.995
T1 (4hours after the first evaluation)	0	8	3.96	2.045
T2 (8hours after the first evaluation)	0	9	3.05	1.890
Control				
T0	3	10	7.54	2.117
T1	1	8	4.04	1.953
T2	1	8	3.61	1.685
Treatment				
T0	4	10	8.52	1.762
T1	0	8	3.89	2.172
T2	0	9	2.48	1.949

Abbreviations: FLACC, face, leg, activity, cry, and consolability; SD, standard deviation.

For the main analysis, a Mann–Whitney U test that was used to evaluate the pain score difference between the control group and the experimental group showed no significant differences between the first evaluation point (T0) and the second evaluation point (T1 =  four hours after the first evaluation point). While for the third evaluation point (T2 =  eight hours after the first evaluation point), there was a significant difference ( *p* = 0.005) for the mean pain score ( Table 3 ).

**Table 3:** Results of the Mann–Whitney U test for significant mean pain score between the control group and the treatment group

Time point	Group	Mean rank	*p* -Value (significant if *p* < 0.05)
T0	Control	24.16	0.63
	Treatment	31.98	
T1	Control	28.79	0.70
	Treatment	27.19	
T2	Control	33.82	0.005
	Treatment	21.96	

As for side effects, no side effects of the curcumin patch in the current study were reported nor detected.

## Discussion

The current study revealed that most of the participants were male participants. This finding is consistent with previous findings, 21 22 23 24 25 of which the male predominance in these types of birth defects was mainly reported. In a literature review by Mairaj et al, it was revealed that male predominance was reported for the prevalence of cleft lip and palate with a male/female sex ratio of 1.81 (CI 95%: 1.75–1.86). 23 In another study conducted by Martelli et al, it was reported that there was a strong association found between the male gender and the presence of clefts (odds ratio = 3.51; CI 95%: 2.83–4.37), and that the majority of infants included in their study were male (61%). 24 The current study also found that most operated cases were those of cleft lips ( Table 1 ), indicating a higher prevalence of this type of cleft. This specific finding is different from those of the previous findings, where the prevalence of cleft lip and palates was higher than those of cleft lips or cleft palates alone. 22 24

In the current study, the difference in pain scores reduction between the control group and the experimental group was detected. This might be due to the effect of the curcumin patch applied in the experimental group. A study conducted by Anil et al on postsurgical patients that underwent a periodontal surgery procedure that evaluated the efficacy of curcumin as an inflammation and analgesic agent through the transmucosal route revealed that postoperative pain scores were significantly reduced in the experimental group. The transmucosal route of drug delivery ensures that the potential therapeutic agent has a maximum contact period of the desired concentration while also ensuring better drug absorption. 26 Another study conducted by Kriplani et al about the efficacy of curcumin-containing patches for the treatment of osteoarthritis also indicated the efficacy of curcumin in providing anti-inflammatory as well as analgesic effects for inflammation of the bone. 27

Additionally, previous evaluations of the effective curcumin dose in humans have been reported. 14 28 29 In these human studies, curcumin was reported to be taken as much as 600 mg per day, 14 500 to 8,000 mg per day for short-term usage and 440 to 2,200 mg per day for long-term usage, 29 and 180 mg per day for consumption period of 6 months. 28 In our study, the dose of the curcumin patch was only 100 mg and was found to be significantly effective in reducing pain scores at 8hours postsurgery. The low yet effective dose of curcumin might be due to the delivery method. Curcumin has been identified for its poor bioavailability due to poor absorption, rapid metabolism, and rapid systemic elimination. 26 Delivery of curcumin through a transdermal route, a similar route to the one used in our study, has been known as a better delivery route to increase curcumin's penetration efficacy. 30 31 It was found that the release of curcumin through this delivery method will increase with time, 30 which might be the underlying explanation for the significant difference in pain scores found on the eighth hour postsurgery.

The significant reduction in pain scores in the experimental group compared with the control group might be due to curcumin's pathway as an analgesic agent. A preclinical study of curcumin showed its efficacy of curcumin in managing acute pain. Curcumin was proven to show antihyperalgesic activity by reversing mechanical hyperalgesia through a dose-dependent manner. Furthermore, when given in a repeated manner, curcumin was also proven to be effective in managing postoperative pain. 16 32 Additionally, postoperative pain has been associated with an increased level of prostaglandin-E2 (PGE2) that is associated with postoperative inflammation. 33 34 Inflammation, which is a natural process following surgery, will induce the release of several inflammation mediators that will then be followed by the infiltration of the inflammatory cells to the damaged site and the activation of nociceptive nerve fibers to produce pain signal. One of the mediators that are increased during the inflammation period of the postoperative healing period is PGE2, 34 35 a mediator that was also found to be increased in patients with postoperative pain. 36 37

This increased level of PGE2 in postoperative patients is of advantage for curcumin's mechanism of action as curcumin is known for its effect in downregulating PGE2. 38 39 The result of previous studies revealed that curcumin affects the arachidonic acid metabolism through the blocking of cytosolic phospholipase phosphorylation, which will then reduce the expression of cyclooxygenase-2 as well as PGE2. 40 41 Furthermore, a study conducted by Sahbaie et al about the efficacy of curcumin in attenuating pain after incision revealed that perioperative curcumin treatment was proven to be effective in attenuating hyperalgesia due to incision when mice were challenged with subsequent application of hind paw PGE2, 42 indicating the direct effect of curcumin on PGE2. Conclusively, this direct mechanism of curcumin on PGE2 is one of the potential underlying mechanisms of the significant difference in pain scores reduction found between the control group and the experimental group in the current study.

### Study Limitation

Even though this study was conducted in a pragmatic method that increases its external validity, several factors have been identified as the limitation of our study. In the current study, considering our hypothesis of the direct effect of curcumin on PGE2, it will be more evident if the PGE2 level was also measured. Therefore, we are currently conducting other studies that involve the measurement of PGE2 in different types of surgery. Hopefully, these ongoing studies will provide better evidence in supporting our current hypothesis of the analgesic potential of curcumin. Additionally, a qualitative study that investigates the effect of curcumin through the caregiver's (medical staff) perspective, as well as the parent's perspective would have added additional value to the study.

## Conclusion

In summary, the current study showed a very promising future for curcumin to be used as an adjuvant analgesic agent for the management of acute orofacial pain due to operative procedures. And considering that our study applied a pragmatic design by following the standardized treatment protocol for acute pain management in postsurgery cleft lip and cleft palate patients, it can be concluded that the external validity of this study is high and therefore, the result is very much applicable in daily practice. Last but not least, considering the direct effect of curcumin on PGE2 found in preclinical studies, it is important to provide further evidence in future clinical studies.

## References

[1] Salari N Darvishi N Heydari M Bokaee S Darvishi F Mohammadi M Global prevalence of cleft palate, cleft lip and cleft palate and lip: a comprehensive systematic review and meta-analysis J Stomatol Oral Maxillofac Surg 2022 123 02 110 120 34033944 10.1016/j.jormas.2021.05.008

[2] Lacerda-Santos R Batista R G Neves S S Effectiveness of secondary alveolar bone graft on canine eruption: systematic review Eur J Dent 2021 15 03 579 587 33622012 10.1055/s-0041-1723070 PMC8382499

[3] Zeraatkar M Ajami S Nadjmi N Faghihi S A Golkari A A qualitative study of children's quality of life in the context of living with cleft lip and palate Pediatric Health Med Ther 2019 10 13 20 30697094 10.2147/PHMT.S173070 PMC6342148

[4] De Cuyper E Dochy F De Leenheer E Van Hoecke H The impact of cleft lip and/or palate on parental quality of life: a pilot study Int J Pediatr Otorhinolaryngol 2019 126 109598 31369974 10.1016/j.ijporl.2019.109598

[5] Vyas T Gupta P Kumar S Gupta R Gupta T Singh H P Cleft of lip and palate: a review J Family Med Prim Care 2020 9 06 2621 2625 32984097 10.4103/jfmpc.jfmpc_472_20 PMC7491837

[6] Zreaqat M H Hassan R Hanoun A Cleft Lip and Palate Management from Birth to Adulthood: An Overview. 2017In: Insights into Various Aspects of Oral Health [Internet]. Intech Open. Accessed April 28, 2022 at:https://www.intechopen.com/chapters/54942

[7] Adu E JK Donkor P Management of cleft lip and palate: a five year review Arch Otolaryngol Rhinol 2017 3 23 26

[8] Augsornwan D Pattangtanang P Pikhunthod K Surakunprapha P Postoperative pain in patients with cleft lip and palate in Srinagarind Hospital J Med Assoc Thai 2011 94(06, Suppl 06)S118 S123 22423426

[9] Bandyopadhyay K H Paul A Postoperative analgesia for cleft lip and palate repair in children J Anaesthesiol Clin Pharmacol 2016 32 01 5 11 27006533 10.4103/0970-9185.175649 PMC4784214

[10] Flowers T Winters R Postoperative pain management in pediatric cleft lip and palate repair Curr Opin Otolaryngol Head Neck Surg 2021 29 04 294 298 34183559 10.1097/MOO.0000000000000719

[11] Peng Z Z Wang Y T Zhang M Z Preemptive analgesic effectiveness of single dose intravenous ibuprofen in infants undergoing cleft palate repair: a randomized controlled trial BMC Pediatr 2021 21 01 466 34674670 10.1186/s12887-021-02907-6 PMC8532298

[12] Rossell-Perry P Romero-Narvaez C Rojas-Sandoval R Gomez-Henao P Delgado-Jimenez M P Marca-Ticona R Is the use of opioids safe after primary cleft palate repair? A systematic review Plast Reconstr Surg Glob Open 2021 9 01 e3355 33564585 10.1097/GOX.0000000000003355 PMC7858197

[13] Mansour R F Abdelghany M S Ultrasound-guided suprazygomatic maxillary nerve block in cleft palate surgery: the efficacy of adding dexmedetomidine to bupivacaine Egypt J Anaesth 2021 37 329 336

[14] Maulina T Diana H Cahyanto A Amaliya A The efficacy of curcumin in managing acute inflammation pain on the post-surgical removal of impacted third molars patients: a randomised controlled trial J Oral Rehabil 2018 45 09 677 683 29908031 10.1111/joor.12679

[15] Zhao G Shi Y Gong C Curcumin exerts antinociceptive effects in cancer-induced bone pain via an endogenous opioid mechanism Front Neurosci 2021 15 696861 34539332 10.3389/fnins.2021.696861 PMC8446608

[16] Basu P Maier C Basu A Effects of curcumin and its different formulations in preclinical and clinical studies of peripheral neuropathic and postoperative pain: a comprehensive review Int J Mol Sci 2021 22 09 4666 33925121 10.3390/ijms22094666 PMC8125634

[17] Rujirachotiwat A Suttamanatwong S Curcumin Promotes collagen type I, keratinocyte growth factor-1, and epidermal growth factor receptor expressions in the in vitro wound healing model of human gingival fibroblasts Eur J Dent 2021 15 01 63 70 33003239 10.1055/s-0040-1715781 PMC7902102

[18] Kriplani P Guarve K Singh Baghel U Formulation optimization and characterization of transdermal film of curcumin by response surface methodology Chin Herb Med 2021 13 274 285 36117499 10.1016/j.chmed.2020.12.001 PMC9476792

[19] Crellin D J Harrison D Santamaria N Babl F E Systematic review of the face, legs, activity, cry and consolability scale for assessing pain in infants and children: is it reliable, valid, and feasible for use? Pain 2015 156 11 2132 2151 26207651 10.1097/j.pain.0000000000000305

[20] Crellin D J Harrison D Santamaria N Huque H Babl F E The psychometric properties of the FLACC scale used to assess procedural pain J Pain 2018 19 08 862 872 29551662 10.1016/j.jpain.2018.02.013

[21] Kim S Kim W J Oh C Kim J C Cleft lip and palate incidence among the live births in the Republic of Korea J Korean Med Sci 2002 17 01 49 52 11850588 10.3346/jkms.2002.17.1.49 PMC3054830

[22] Yılmaz H N Özbilen EÖ Üstün T The prevalence of cleft lip and palate patients: a single-center experience for 17 years Turk J Orthod 2019 32 03 139 144 31565688 10.5152/TurkJOrthod.2019.18094 PMC6756567

[23] Mairaj K A Bui A H Taioli E Epidemiology of Cleft Lip and Palate, Designing Strategies for Cleft Lip and Palate Care. 2017. Intech OpenAccessed April 28, 2022 at:https://www.intechopen.com/chapters/53918

[24] Martelli D R Coletta R D Oliveira E A Association between maternal smoking, gender, and cleft lip and palate Rev Bras Otorrinolaringol (Engl Ed) 2015 81 05 514 519 10.1016/j.bjorl.2015.07.011 PMC9449023 26277833

[25] Pool S MW der Lek L MV de Jong K Vermeij-Keers C Mouës-Vink C M Embryologically based classification specifies gender differences in the prevalence of orofacial cleft subphenotypes Cleft Palate Craniofac J 2021 58 01 54 60 32602363 10.1177/1055665620935363 PMC7739112

[26] Anil A Gujjari S K Venkatesh M P Evaluation of a curcumin-containing mucoadhesive film for periodontal postsurgical pain control J Indian Soc Periodontol 2019 23 05 461 468 31543620 10.4103/jisp.jisp_700_18 PMC6737849

[27] Kriplani P Guarve K Baghel U S Novel herbal topical patch containing curcumin and Arnica montana for the treatment of osteoarthritis Curr Rheumatol Rev 2020 16 01 43 60 30767746 10.2174/1573397115666190214164407

[28] Nakagawa Y Mukai S Yamada S The Efficacy and safety of highly-bioavailable curcumin for treating knee osteoarthritis: a 6-month open-labeled prospective study Clin Med Insights Arthritis Musculoskelet Disord 2020 13 1.179544120948471E15 10.1177/1179544120948471 PMC7425263 32848491

[29] Daily J W Yang M Park S Efficacy of turmeric extracts and curcumin for alleviating the symptoms of joint arthritis: a systematic review and meta-analysis of randomized clinical trials J Med Food 2016 19 08 717 729 27533649 10.1089/jmf.2016.3705 PMC5003001

[30] Eckert R W Wiemann S Keck C M Improved dermal and transdermal delivery of curcumin with SmartFilms and nanocrystals Molecules 2021 26 06 1633 33804137 10.3390/molecules26061633 PMC8000619

[31] Sintov A C Transdermal delivery of curcumin via microemulsion Int J Pharm 2015 481(1-2):97 103 25655717 10.1016/j.ijpharm.2015.02.005

[32] Zhu Q Sun Y Yun X Ou Y Zhang W Li J X Antinociceptive effects of curcumin in a rat model of postoperative pain Sci Rep 2014 4 4932 24816565 10.1038/srep04932 PMC4017214

[33] Li Q B Chang L Ye F Luo Q H Tao Y X Shu H H Role of spinal cyclooxygenase-2 and prostaglandin E2 in fentanyl-induced hyperalgesia in rats Br J Anaesth 2018 120 04 827 835 29576123 10.1016/j.bja.2017.11.103 PMC6200103

[34] Al-Waeli H Nicolau B Stone L Chronotherapy of non-steroidal anti-inflammatory drugs may enhance postoperative recovery Sci Rep 2020 10 01 468 31949183 10.1038/s41598-019-57215-y PMC6965200

[35] Attiq A Jalil J Husain K Ahmad W Raging the war against inflammation with natural products Front Pharmacol 2018 9 976 30245627 10.3389/fphar.2018.00976 PMC6137277

[36] Grga D Dzeletović B Damjanov M Hajduković-Dragojlović L Prostaglandin E2 in apical tissue fluid and postoperative pain in intact and teeth with large restorations in two endodontic treatment visits Srp Arh Celok Lek 2013 141(1-2):17 21 23539905 10.2298/sarh1302017g

[37] Si H B Yang T M Zeng Y Correlations between inflammatory cytokines, muscle damage markers and acute postoperative pain following primary total knee arthroplasty BMC Musculoskelet Disord 2017 18 01 265 28623906 10.1186/s12891-017-1597-y PMC5473999

[38] Mohd Aluwi M FF Rullah K Haque M A Suppression of PGE2 production via disruption of MAPK phosphorylation by unsymmetrical dicarbonyl curcumin derivatives Med Chem Res 2017 26 3323 3335

[39] Pearson W Kott L S A biological extract of turmeric (Curcuma longa) modulates response of cartilage explants to lipopolysaccharide BMC Complement Altern Med 2019 19 01 252 31506082 10.1186/s12906-019-2660-z PMC6737590

[40] Dasuni Wasana P W Vajragupta O Rojsitthisak P Towiwat P Mechanistic insight into the effects of curcumin on neuroinflammation-driven chronic pain Pharmaceuticals (Basel) 2021 14 08 777 34451874 10.3390/ph14080777 PMC8397941

[41] Uddin S J Hasan M F Afroz M Curcumin and its multi-target function against pain and inflammation: an update of pre-clinical data Curr Drug Targets 2021 22 06 656 671 32981501 10.2174/1389450121666200925150022

[42] Sahbaie P Sun Y Liang D-Y Shi X-Y Clark J D Curcumin treatment attenuates pain and enhances functional recovery after incision Anesth Analg 2014 118 06 1336 1344 24755847 10.1213/ANE.0000000000000189

